# Annual Meteorological Table for Sidmouth, Devon, 50° 41′ N. Lat. 3° 13′ W. Long

**Published:** 1815-03

**Authors:** 


					For the Medical and Physical Journal.
Annual Meteorological Table for Sidmouth, Devon, 50? 41' N. laf. 3? 13' W. long;.
by Dn. Clarke.
Vhermorneter?(Northerly exposure.
X bc
1 &
29 44? if? 21
8 53 nw 21
27 54 svv 7
13 63 n 4
26 67 s 11
75 s 7
25 76 se 2
4 76 s
1 73 s 10
14 61 s 9
14 57 n 21
111 57 sw 26
?O
co <l>
e;
*
?3 5
16?
23
27
35
38
43
47
46
42
33
31?
38
40
'19
52
59
61
61
57
46
nw 41
uj 42
? _C
c tsJ
PJ
o ?
s 2
35?
43
44
56
61
66
71
70
66
55
49
48
29?
33
35
43
44
51
55
51
50
44
39
40
?? 5
s
|5
? 4-?
32
38
39
50
5 2
58
63
62
58
50
44
44
O E'
a "o
43 46
33?
39
40
52
54
61
67
65
62
52
45
44
31?
36
37
45
46
52
56
55
52
46
43
42
e e.
? Q
TH
O
? E
u .
go
33?
38
39
50
51
58
63
62
58
50
44
43
tS
s.??
o
15?
12
10
8
10
10
9
10
9
12
11
17
1 30-21
30-51
30 50
30-3*
*0-51
30-41
30-36
30-39
30*41
30-34
30-44
30 31
28-50
29-83
28-96
29-4*
29-41
29-76
29-74
29-70
29-70
29-19
29-36
29-12:
5 E
V
22
30.14
29-86
29-92
30 04
30 10,
3004
30 08
30-15
29-87
29-85
29-79
o ??
Hygrometer of De Luc. Weatne|. Wm'cs.
41?
40
39
30 42
aw 10
2
21
sw| 16
16 1 14 4
20 1 7 9 II
1? 2 12 IS 8 5
15 l 14 4 7 ll
20 10 1 6 14 9
18 1 11 5 9 4
20 1 10 1 4 12
16 1 14 "4 2 20
14? sw 15" 25? 20 0 10 8 910
14 Nt 13 28 17 0 1J 1 H H 11
25 nw 10 31 15 1 14 C 0 .814
25 nw 14 35 12 0 1915 4 t210
ANNUAL RESULTS.
Thermometer.
Highest by Six's Register, 25th July 76?
Lowest by ditto in the night, 21st Jan. ???? 18
Mean of these extremes ?     48
Mean of the mean of the highest. ..?,???< 5,5
Mean of the mean of the lowest  43
Mean of these two ? ? ?    49
Mean of 9 morning     49
Mean of 2 afternoon     53
Mean of 9 morning and 2 afternoon  51
Mean of 11 at night  45
Mean of 9 a. m. ? p. m. and 11 p.   49
Greatest variation in 24 hours, Dec. 19-20 ,, 17
Mean temperatures from
observations taken at ? ? 9 a.m. \
2p.rn.8f
11 p.m.
For Nov. Dec. and Jan. ? ? 40
? Feb. March, and April 43
? May, June, and July 58
? August, Sept. and Oct. 57
??Six winter months ?? 42
? Six summer months ?? 58
Annual mean    50
Barometer.
Highest obs. 17th Feb.?.?? 30*51
Lowest ? 29th Jan. ? ? ? ? 28*50
Greatest range, Jan. 28-29 *91
Annual mean  29 95
De Luc's Ftygrometer.
Mean for the months 30?.
Weather, days.
Fine ???? 206
Cloudy ?? 19
Wet 140
365
Wind. days.
N and NE 81
E a fid ?E 77
S and SW 107
W and NW 100
365
Rain for the year, 25 in. 73 dec.
Mr. Machell's Description of his Annular Saw. 19&
From the Editor.?We shall be thankful to the ingeuious au-
thor of the foregoing Table if he can, on a future occasion, favour
us with the progress of diseases, and, as far as his observation
extends, their connection with the state of the weather.

				

